# Evaluation of biocementation of slope soil for erosion control with low-cost materials

**DOI:** 10.1038/s41598-024-67185-5

**Published:** 2024-07-11

**Authors:** M. Azizul Moqsud, Takuya Gochi

**Affiliations:** Department of Civil and Environmental Engineering, Yamaguchi University, Ube City, Japan

**Keywords:** Biocementation, Erosion control, Low-cost, Slope soil, Rainfall, Environmental sciences, Engineering

## Abstract

In this study, biocementation of slope soil was performed using low-cost, commercially available materials to create a nutrient solution with native *Cytobacillus hornekea*. The high cost of laboratory-grade materials and microbes for biocementation is one of the main obstacles to its popularity. However, the cost of biocementation has been reduced significantly without reducing the strength when low-cost materials were used instead of laboratory-grade materials in this study. Direct shear test results and SEM also proved the suitability of the low-cost biocementation. Artificial rainfall with an intensity of 50–60 mm/h resulted in soil erosion of around 10% and 2% without and with biocementation, respectively. The amount of produced calcium carbonate was around 3.9% while using the low-cost materials with native microbes which is quite comparable with the laboratory-grade materials (3.4%).

## Introduction

Biocementation is a promising ecological method that helps stabilize soil and increase its strength. It is based on the microbial-induced carbonate precipitation (MICP) process that results in the accumulation of calcium carbonate. This method is gaining increasing interest among geotechnical engineers worldwide^1–4^. In the biocementation process, bacterial species present in nature precipitate calcium carbonates through several mechanisms^5–7^. Although it may seem like an issue, the process of stabilizing soil and improving its strength is a natural one. In recent times, Microbially Induced Calcium Carbonate Precipitation (MICP) has gained popularity among researchers as a solution to geological disasters such as landslides and liquefaction^8–10^. The MICP method involves using calcium chloride as a source of calcium for the bacteria to metabolize, which in turn leads to the generation of calcium carbonate. The microbes act in an additional important role: because the cell surfaces of bacteria are negatively charged, the Ca^2+^ ions are attracted, and nucleation for calcium carbonate precipitation starts on these surfaces^11–15^. Amongst the extensive variety of uses of MICP, soil stabilization in the slope is getting increased consideration as slopes are frequently associated with human interaction such as highway systems and residential areas^16–19^. For the stability of slope soil, surface erosion is a great challenge^20–22^. Especially, rainfall-induced slope disaster is a common problem worldwide. A high rate of rainwater infiltration is generally liable for slope erosion and slope failure^21^. The traditional method such as cement stabilization is not environmentally friendly as the chemical cement industries discharge around 8% of the total greenhouse gases during their production. On the other hand, in the biocementation process, carbon is captured and used to produce calcium carbonate. So, biocementation could be an alternate of chemical cement in the future in some applications of geotechnical engineering works. *Sporosarcina pasteurii* is one of the most studied bacteria: it empowers an extremely active urease enzyme associated with the hydrolysis of urea^23,24^. The strengthening of sand using *S. pasteurii* can control surface erosion of the slope^25–27^ and decrease hydraulic conductivity while increasing soil strength^28^. As per references^29,30^, it has been conclusively demonstrated that *S. pasteurii* possesses the ability to form hard surfaces^31^. The cost of the materials for biocementation is a challenge and one of the main hindrances to its practical application in the real field. The majority of previous MICP studies have relied on using expensive laboratory-grade nutrient media such as yeast extract, nutrient broth, and soy broth. However, some recent studies have explored using alternative, less expensive nutrient sources like corn steep liquor^32^, chicken manure effluent, lactose mother liquor, beer-yeast, and food-grade yeast to grow various ureolytic bacterial species^33–36^. The results of these studies have shown that the growth and performance of the species cultivated in alternative media are comparable to those grown in conventional media. Previous studies have also attempted to evaluate the suitability of using alternative calcium and urea sources instead of laboratory-graded chemicals. For example, eggshells and calcareous sand were proposed as a replacement for synthetic calcium chloride^37,38^, and researchers found that these calcium-rich materials can be dissolved in acid to produce the required soluble calcium for MICP purposes. More recently, Chen et al.^39^ discovered that pig urine, which is rich in urea, can be used for MICP treatment instead of analytical-grade urea. The use of waste materials has two significant advantages: (1) it reduces the material cost of the treatment and (2) it contributes to waste mitigation. Unfortunately, most contemporary MICP studies still use expensive analytical-grade chemicals for cementation media, which limits the technology’s progress. Therefore, it is essential to introduce more alternative sources that are (1) low-cost or no-cost, (2) available, and (3) accessible to replace synthetic calcium and/or urea sources.

Currently, laboratory-grade nutrient solutions need around 250 (500 ml) Japanese Yen to prepare, however, in this proposed study with low-cost materials, it can be reduced to only 15 Japanese Yen. A study investigated the use of plant extracts as a low-cost alternative to laboratory-grade materials for biocementation^34^. The study showed that plant extracts can effectively promote MICP and improve the strength of the soil. Similarly, Wang et al.^25^ explored the use of waste materials such as fly ash and phosphogypsum as low-cost alternatives for biocementation. A previous study showed that the use of waste materials can effectively promote MICP and improve the strength of the soil. Overall, the literature suggests that low-cost biocementation can be a promising solution to reduce the cost of soil stabilization without compromising its strength. However, easily available low-cost materials for nutrient solutions that are commercially sold in the market have not been found so far.

The main objective of this research is to investigate the effectiveness of low-cost materials (both easily and commercially available) for biocementation in stabilizing natural slope soil and compare it with the performance of laboratory-grade materials when used with the native bacteria *Cytobacillus horneaka*. Additionally, the study intends to assess the impact of rainfall on the biocemented slope soil, using surface erosion measurement and image analysis as evaluation tools. The reason of choosing *Cytobacillus horneaka* instead of *S. pasteurii* is that *Cytobacillus horneaka* is a native species in Japan and it will reduce the cost of the bacteria and easy to adopt with the weather conditions in Japan.

## Materials and methods

The soil sample was collected from the natural slope of the Yamaguchi University, Ube campus (33.9431° N, 131.251° E), Japan. The soil collected from the natural slope contains a water content of 10% and is classified as sandy soil after the grain size analysis^16^.The soil sample was cleaned by removing the roots and other visible ingredients such as stones and dried leaves (by sieving) and used for the isolation of ureolytic native bacteria which are responsible for the biocementation. In this case, the *Cytobacilus horneaka* has been isolated and grown in the incubator to increase the number. The previous study assessed urease activity in this bacterium, and the results were used in this study, as described by Moqsud and Gochi^16^. In brief, The culture was maintained in a shaker at 30 °C and 160 rpm, and the growth curves were obtained by monitoring the solutions’ optical density at the wavelength of 600 nm (OD600) with time. The urease activity of *Cytobacilus horneaka* is 12 U/ml. The chemicals used for preparing the culture liquid and the nutrient liquid are shown in Tables 1 and 2. To reduce the cost of materials for biocementation, low-cost and easily available materials have been used rather than laboratory-grade pure chemicals. The comparison of the cost of the two types of nutrient solution is shown in Table 3. No nutrient broth was used for low-cost biocementation. It has been observed that the low-cost material shows a dramatic cost reduction. Two types of nutrient solutions have been prepared with laboratory-grade pure materials and low-cost easily available materials, respectively to compare the effectiveness of the low-cost materials. For laboratory-grade materials urea and calcium chloride (Wako Chemicals Corporation) were used which are 100% pure and generally used in standard laboratory experiments. The low-cost urea (Akagi Engei Corporation) is commercially available and collected from the fertilizer shop which is generally used by the farmers and the purity is around 97% mixing with other nutrients such as potassium which is good for plants. The commercially available CaCl_2_ (Sun and Hope Corporation) was collected from the shop which is used as a snow-melting agent. The purity is around 97%. Both of these chemicals are widely used and do not have adverse effects on the geo-environmental condition and the soil microbes. Distilled water is used for preparing the culture and nutrient solutions. To avoid the effect of temperature on biocementation, all the experiments were conducted at a constant room temperature of 25 °C. The duration of nutrient treatment was selected as 10 days based on the previous studies and each day the same amount of nutrient solution was applied^16,41,42^.

**Table 1:** Constituents of culture solutions.

Materials	Amount
Peptone	5 g
Ammonium sulfate	5 g
1.03 M tris	7.87 g
Yeast extract	10 g
Distilled water	500 ml

**Table 2:** Constituents of nutrient solutions.

Materials	Amount
Urea	15 g
Calcium chloride	27.75 g
Distilled water	500 ml

**Table 3:** Comparison of cost between low-cost and laboratory-grade nutrient solution.

Material	Laboratory-grade	Low-cost
Urea (15g)	42.90 Yen	9.68Yen
Calcium chloride (27.75 g)	138.20 Yen	4.98 Yen
Nutrient Broth (5g)	59.07 Yen	–
Total	240.17 Yen	14 Yen

### Direct shear test

**Figure 1:**
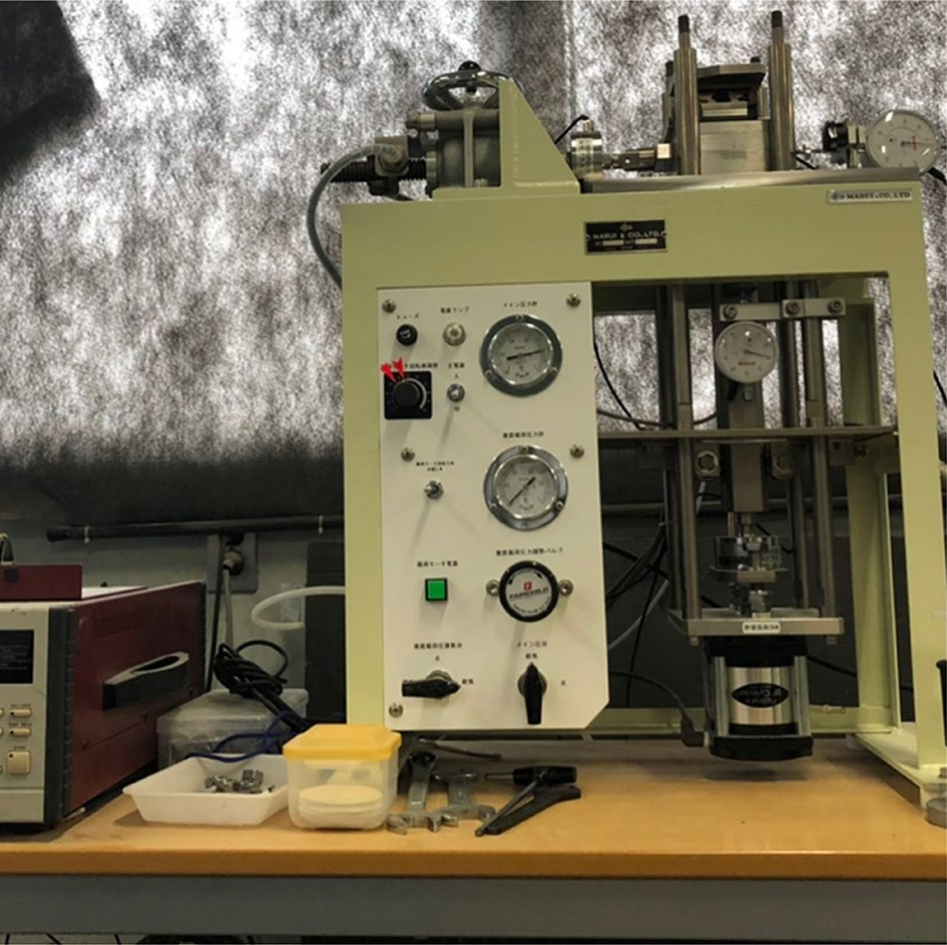
Direct shear test apparatus used in biocementation.

A direct shear test has been carried out to measure the soil strength by using ASTM D3080-04. A 60 × 20 mm cylindrical mold has been used for the direct shear test. The constant pressure one directional shear test has been conducted with 50,100 and 150 kN/m^2^ confined pressure which is shown in Fig. 1. The rate of the shear was fixed at 0.2 mm/min. Three samples have been prepared for the direct shear test by placing slope soil in three layers in the cylindrical mold and compacting with a hammer 25 times in each layer; one with laboratory-grade materials, another one is low-cost materials, and the last one without any biocementation (blank sample which is untreated) ^43–45^. All the specimens were prepared with the same relative density of 85%. The reason behind this density is that the embankment slopes are generally designed with 85% density. ASTM standard D4254 was used to measure and prepare the soil samples with mentioned density Based on the Unified Soil Classification System (USCS) (ASTM 2017), the soil can be classified as poorly graded (SP).

### Scanning electron microscope (SEM) image

The main objective of SEM analysis is to observe the surface of the soil particles after the biocementation and compare them with the blank samples. SEM analysis can reveal the production of calcite on the surface of the soil particles and has become a popular option among bio geotechnical engineers^16,46–48^.

SEM photos were taken by using a JEOL JSM-7900F field emission scanning electron microscope. Before SEM photos were taken, cemented specimens were dried for 24 h in the oven at 110 °C^16^. Tests were compiled from bio-cemented and non-cemented soil specimens.

### Effect of rainfall on biocemented slope

**Figure 2:**
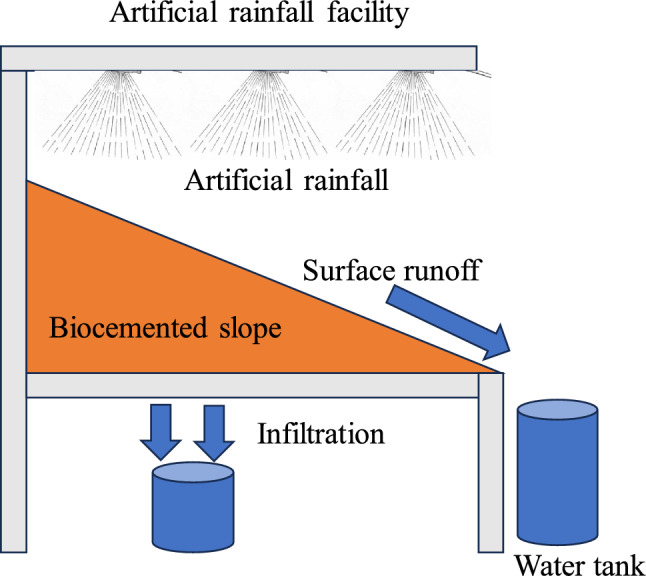
Schematic diagram of artificial rainfall experiment on biocemented slope by low-cost materials.

Figure 2 illustrates the schematic diagram of the artificial rain experiment on the biocemented and non-biocemented slopes. Artificial rainfall of 50–60 mm/h intensity with three different slope angles (30°, 35°, 40°) has been applied on the biocemented slope (120 × 60 × 5 cm). The intensity of this rainfall was chosen because of the recent (2022) rainfall-induced slope disasters that happened in Hiroshima (Japan) with the mentioned rainfall intensity. Another slope was prepared without biocementation and the same amount of rainfall was applied to compare the effect of rainfall on the biocemented slope. The slope was prepared with the same density as the direct shear test experiment and the same nutrient solution was applied for the same number of days (10 days). The reason behind the 10-day treatment was that it could be biocemented properly and uniformly throughout the slope which has been revealed in previous studies^16^. The biocemented slope with different slope angles was examined in the laboratory. For the collection of eroded soil, surface runoff was collected, and oven-dried to get the data for erosion. The infiltration of rainwater through the slope was also monitored and collected to check the soil erosion under the slope as shown in Fig. 2.

### Calcium carbonate determination

The amount of calcium carbonate produced in soil due to biocementation was determined by using the method of calcium carbonate determination by using ASTM standard D4373-14. This method serves as an index for the carbonate content of soil. The method is gasometric, utilizing a simple portable apparatus and hydrochloric acid according to the ASTM standard. The details of the methods are described in the standard.

## Result and discussions

**Figure 3:**
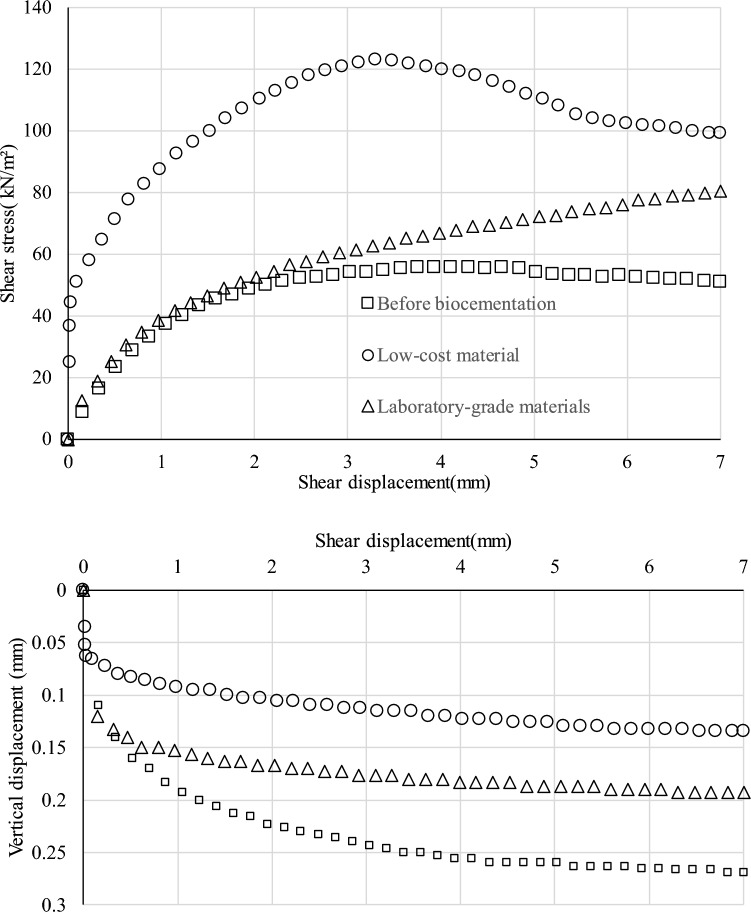
Result of the direct shear test for 100 kN/m^2^ confining pressure with shear displacement in the top and the vertical displacement in the bottom.

Figure 3 illustrates the variation of shear stress with shear displacement for a confining pressure of 100 kN/m^2^ for all three samples. The result shows that the strength of the natural slope soil has increased significantly due to biocementation. This is because low-cost materials, when combined with the metabolic action of bacteria, can increase the strength of natural soil. The interesting thing is that the increase of strength is more prominent in low-cost biocementation compared to laboratory-graded materials. It was observed that the amount of generated calcium carbonate was higher for low-cost materials (3.9%) than for laboratory-grade materials (3.4). In this experiment, the total amount of calcium carbonate was measured through the chemical analysis (ASTM D 4373-14). Furthermore, the results show that the vertical displacement value was lower after biocementation than before.

**Figure 4:**
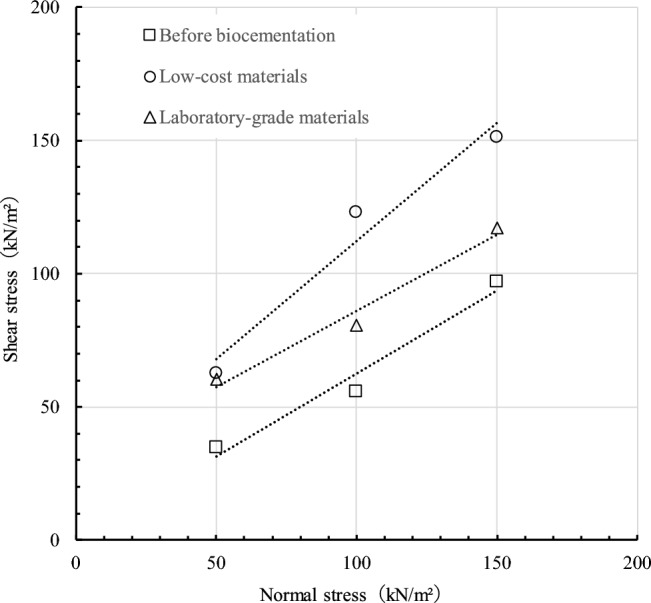
Relation between shear stress and normal stress for laboratory grade, low-cost and without biocementation specimen.

Previous research has found that using eggshells instead of calcium chloride results in a higher UCS (unconfined compressive strength) of 649.7 kPa, which is about 29% higher than samples treated with pure-grade calcium chloride^36,49^. This is because eggshells have higher calcium content. In another study, industrial-grade calcium chloride and urea were found to result in a lower surface UCS (1.45 MPa) compared to laboratory-graded chemicals (1.79 MPa)^9,38,39,50,51^. Figure 4 shows the relation between shear stress and normal stress. The results revealed that strength increased after the biocementation both for the laboratory-grade and low-cost materials. The angle of shear resistance and cohesion of the soil improved significantly after the biocementation. The bonding between the soil particles increased with the generation of the calcium carbonate generation^39–42,49^. Figure 4 also shows the value of cohesion and the angle of friction for all three specimens. The cohesion and angle of friction were 0.1, 28.9, 23.6 kN/m^2^, and 35.8°, 32.7°, and 50.8°, for blank, laboratory-grade, and low-cost samples, respectively. It is confirmed that after biocementation, the stiffness increased compared to without biocementation. A similar type of result was observed with other researchers^50,51^^.^

**Figure 5:**
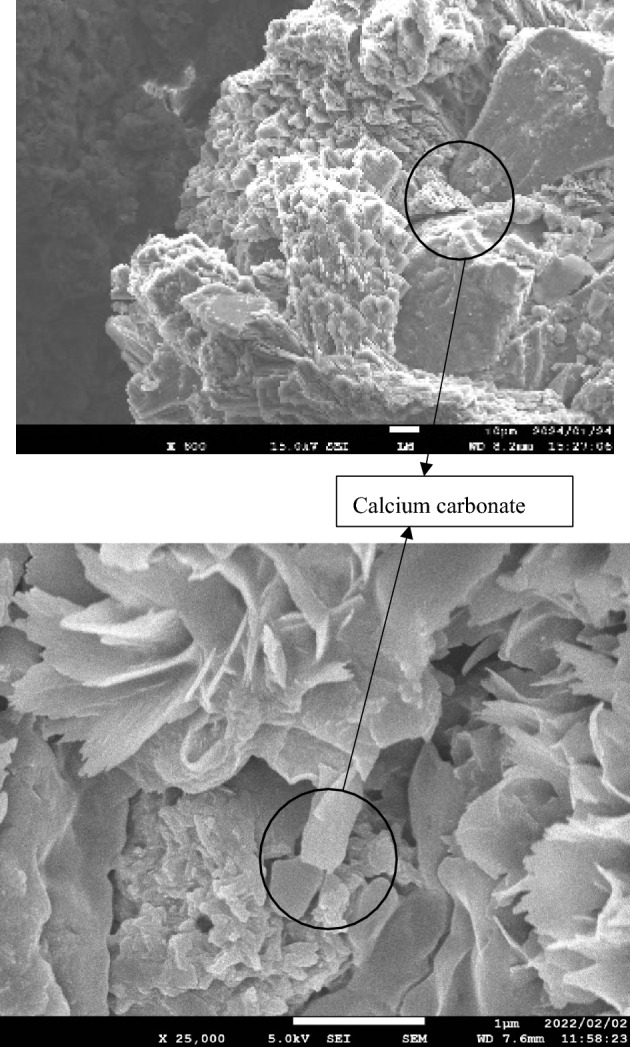
Scanning electron microscopic photo of biocemented soil with low-cost, laboratory-grade (from top and bottom, receptively).

Figure 5 shows the scanning electron microscope (SEM) photo of the biocemented soil sample. Calcium carbonate has precipitated on the entire surface of the soil particles both laboratory-grade and low-cost materials^46–48^. The difference in the calcium carbonate produced on the surface is not observed in both types of materials. Calcium carbonate which has been precipitated on the surface of the soil particles can create the bonding among them. This bonding is the main source of increasing strength of the slope soil and consequently reduces the erosion and infiltration of rainwater. This proof of biocementation has gained significant attraction among researchers worldwide^1,16,19,50,51^.

Previous research has shown that the increase in shear strength of sand is directly related to the amount of calcium carbonate present in the range of 1–2.5%^47,48,52^. As the calcium carbonate content exceeds 2.5%, the improvement in strength becomes less significant because nearly all available particle contact points are already bonded by CaCO_3_^35,49^. The increase in calcium carbonate content significantly affected the shear strength of biocemented soil by increasing soil cohesion, while the friction angle was not significantly affected by the cementation process. When the highest shear resistance is reached, local shearing can initiate the breakage of bonds at particle–particle contacts, leading to the loss of effective cementation. This could be the reason for the current result observed in the laboratory-grade and commercial materials used for biocementation as shown in Fig. 3. However, further studies are needed to gain a better understanding of this phenomenon.

**Figure 6:**
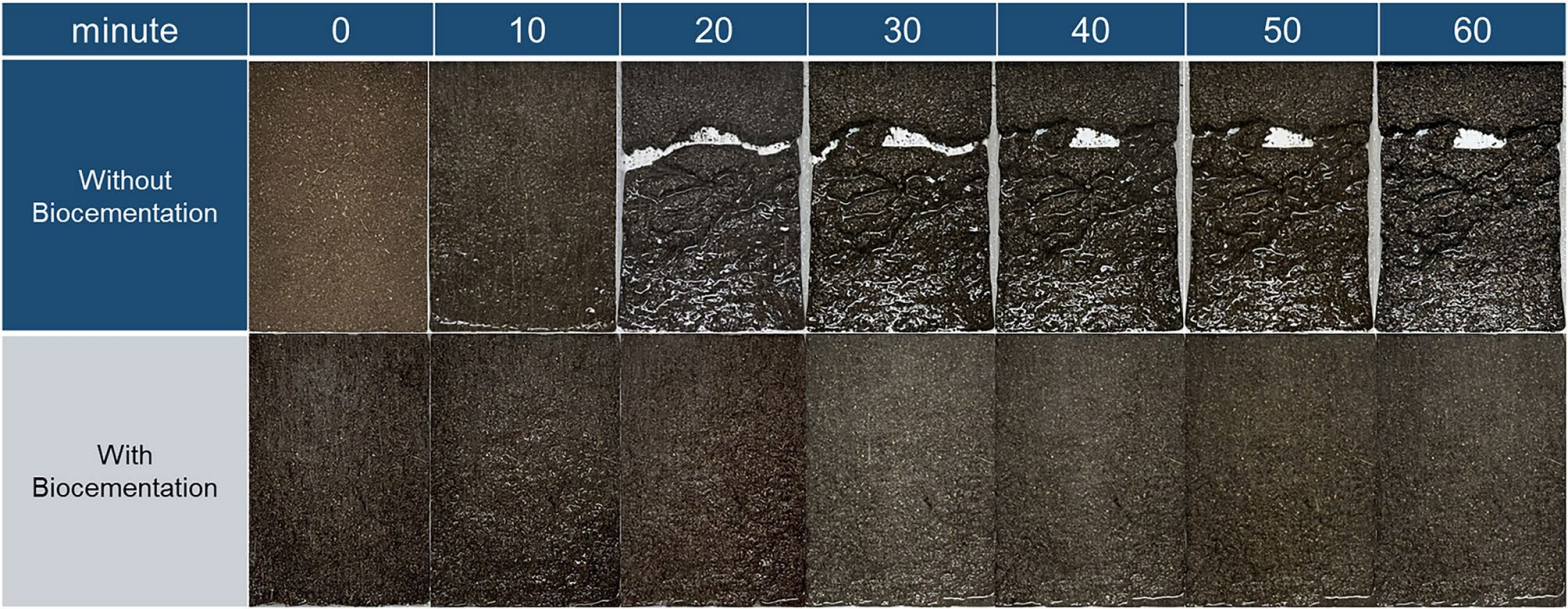
Effect of rainfall on without biocemented slope and biocemented slope.

In Fig. 6, it is observed that a comparison of two photos of a sloped surface—one with biocementation and the other without—at different durations of rainfall, ranging from 0 to 60 min. The observations show that over time, the upper portion of the slope began to collapse, particularly after 10–20 min of rainfall. The slope without biocementation showed significant failure after an hour of rainfall, while the biocemented slope remained stable, proving the effectiveness of biocementation in preventing slope erosion due to rainfall. This study highlights the importance of using low-cost nutrient solutions to increase the strength of soil and promote bonding among soil particles, thus preventing slope failure and surface erosion. The bonding created by the nutrient solution is responsible for the stability of the biocemented slope, which did not collapse even after an hour of continuous rainfall. In conclusion, these findings demonstrate the potential benefits of biocementation in preventing slope erosion, which is a critical issue in construction and infrastructure projects. By using low-cost materials, biocementation can be an affordable and sustainable solution to prevent slope failure and surface erosion, ensuring the longevity and safety of infrastructure projects.

**Figure 7:**
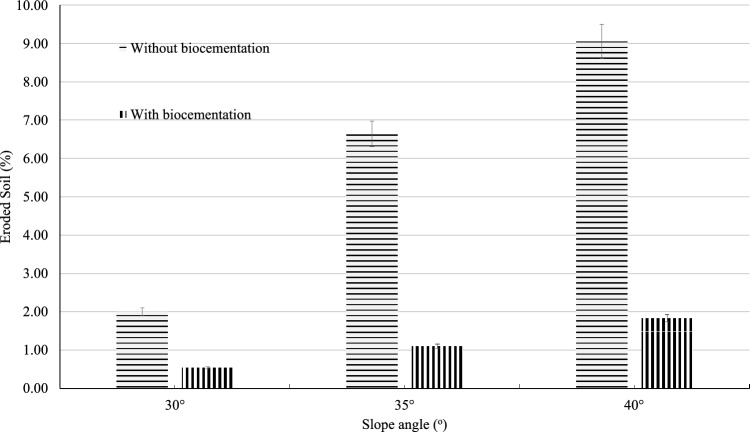
Soil erosion on biocemented and without biocemented slope.

Figure 7 displays a comparison of the erosion amount between biocemented and non-biocemented soil at varying slope angles after a rainfall experiment. The experiment lasted for 60 min, and the collected soil samples included those from surface runoff and infiltration. It has been discovered that after implementing biocementation on a slope, the percentage of eroded soil decreases significantly compared to slopes without it. The amount of erosion is also higher when the slope angle is steeper. The highest amount of erosion was found on a 40-degree slope, with around 9.05% (by weight) of soil eroded without biocementation, and only 1.72% with biocementation. Additionally, the amount of infiltration was smaller on the biocemented slope (250 ml) compared to the uncemented slope (600 ml). The hydraulic conductivity of the slope soil has reduced due to the generation of calcium carbonate between the soil particles and hence reduce the infiltration of rainwater. So, the infiltration amount can also be an indicator of biocementation by using the low-cost materials.

**Figure 8:**
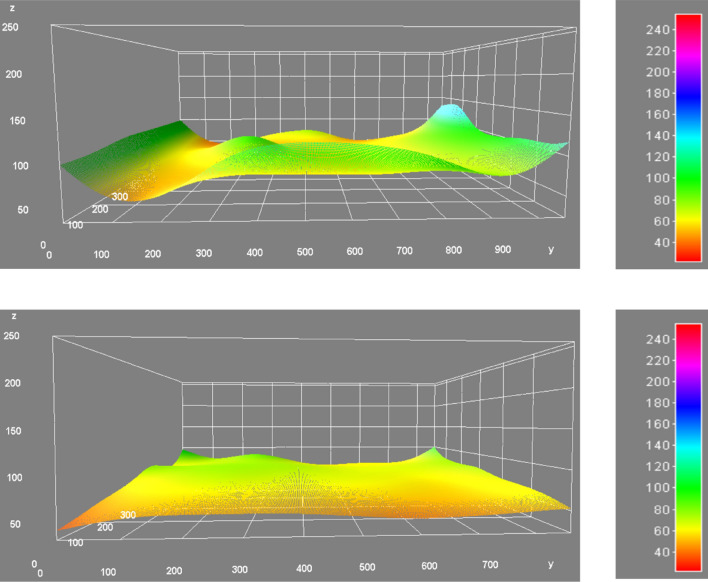
Image analysis (imageJ) of the surface of the slope without biocemented (top) and with biocemented (bottom) after the rainfall experiment (60 min).

Figure 8 shows the image analysis of the surface of the uncemented and biocemented slope (with low-cost materials) after the rainfall (60 min). The image analysis has been done with imageJ software. It has clearly shown that the surface of the uncemented has been eroded with the effect of rainfall after 20 min of rainfall with an intensity of 60 mm/h. However, the surface of the biocemented slope did show much distortion even after the artificial rainfall for 60 min. The intensity of rainfall might be the key factor in this research. However, the rainfall intensity of 60 mm/h has been chosen from the recent (2022) landslide disasters in the Hiroshima prefecture (Japan) due to heavy rainfall. The rainfall-induced surface erosion has also seen a similar trend in previous studies^16^.

Chemical analysis was conducted (ASTM D4373) of the biocemented soil to measure the amount of calcium carbonate with low-cost materials. The analysis revealed that approximately 3–4% of calcium carbonate was produced in both cylindrical samples for direct shear tests and the slope soils for rainfall experiments after 10 days of nutrient treatment (10 days). The range of produced calcium carbonate depends on various factors including the type of microbes, temperature, and nutrient supply which has been shown previously by other researchers^47–50^. In this research, the amount of calcium carbonate generation was observed in low-cost and laboratory-grade materials was around 3.9 and 3.4%, respectively.

Overall, the use of low-cost materials in biocementation has the potential to make this eco-friendly technique more accessible and feasible for slope stabilization^52^. However, further research is needed to optimize the use of these materials and to evaluate their long-term effectiveness in different soil types and environmental conditions.

## Conclusions

In recent years, slope erosion caused by heavy rainfall has become a growing concern in various places across the globe. In this study, the possibility of using the MICP method for stabilizing the slope soil to mitigate the erosion potential has been conducted involving low-cost nutrient materials and native bacteria. To make this novel technology easy to use, the commercially available materials will be used rather than laboratory-grade pure chemicals resulting in a substantial improvement of strength by using native bacteria called *Cytobacillus horneaka*. The low-cost chemicals can reduce the total cost of materials for biocementation significantly (almost 90%). The calcium carbonate generated by low-cost commercial materials showed a remarkable amount (3.9%) so that it can be used in the field revealed in SEM and chemical analysis. The direct shear test also showed that the strength of the low-cost commercial chemicals can increase the strength of the natural slope soil. The increase in slope angle led to a rise in soil erosion. The maximum amount of erosion occurred at a 40-degree slope, with the eroded soil accounting for approximately 9.05% without biocementation, and 1.72% with it. To summarize, affordable materials possess the capability to enhance the soil strength of slopes to a level comparable to that of laboratory-grade materials. This could soon make biocementation a feasible option for large-scale field application of this innovative technology.

## Data Availability

The datasets used and/or analyzed during the current study are available from the corresponding author upon reasonable request.
